# A 0.6-V All-Digital Temperature Sensor with Reduced Supply Sensitivity

**DOI:** 10.3390/s25237181

**Published:** 2025-11-25

**Authors:** Hui Zhou, Yi Wang, Shuang Xie

**Affiliations:** 1 School of Integrated Circuits, Shandong University, Jinan 250100, China; 2 Academy of Intelligent Innovation, Shandong University, Jinan 250100, China; 3 Quan Cheng Laboratory, Jinan 250100, China

**Keywords:** temperature sensor, supply sensitivity, low voltage supply, two-point calibration, all-digital

## Abstract

The present work introduces a 0.6-volt, all-digital, synthesizable temperature sensor characterized by reduced sensitivity to supply voltage variations. The design incorporates two distinct logic delay lines that are distinguished by their equivalent transistor lengths. These variations in transistor lengths result in varying threshold voltages and thermal dependencies. The difference in thermal dependency is detected through the ratio of their charging currents, which are subsequently transformed into digital outputs via their propagation delays. By employing two types of delay lines, the sensor achieves an eightfold reduction in power supply sensitivity compared to configurations utilizing a single delay line and also obviates the necessity for an external clock. Fabricated with 55 nm CMOS technology, the proposed sensor exhibits an inaccuracy of ±1 °C, evaluated through global linear fitting and two-point calibration across five chips, within a temperature range of 20 to 90 °C. The all-digital temperature sensor consumes 2 nanojoules (nJ) for each conversion, with a conversion duration of 0.8 milliseconds (ms) and a resolution of 0.2 °C. The prototype’s physical dimensions are 37 × 31 ${\mu m}^{2}$. Additionally, synthesis on a Cyclone IV FPGA reveals similar characteristics in terms of supply sensitivity reduction.

## 1. Introduction

The power consumption of microprocessors is experiencing a continual increase, correlating with advancements in processing speed and technology scaling. For instance, the Intel i9’s 14900k microprocessor exhibits a Maximum Turbo Power (MTP) of 253 W, surpassing the i7-13700’s MTP of 219 W [1,2]. As documented in [3], each microprocessor is equipped with multiple Digital Temperature Sensors (DTSs), which provide relative temperature data rather than absolute measurements. This relative data enables dynamic adjustments to the cooling fan’s speed, potentially triggering alerts to prevent the microprocessor from exceeding its specified temperature threshold. For optimal thermal monitoring and management, it is crucial that these DTSs are positioned adjacent to thermal hotspots. Consequently, they must be scalable in accordance with the microprocessor technology node while incurring minimal area and power overhead. Furthermore, it is advisable to avoid the use of bias or reference circuits [4]. To fulfill these requirements, temperature sensors based on logic delay lines or voltage-controlled oscillators (VCOs) are preferred [4,5,6,7,8,9,10,11,12]. In particular, sensors constructed entirely from digital logic cells can be synthesized on platforms such as FPGAs [12]. However, as highlighted by Anand [4], temperature sensors that use delay lines or VCOs are susceptible to supply voltage sensitivity. To mitigate this sensitivity, Anand [4] suggests employing dual voltage-controlled oscillators (VCOs). The configuration involves a loading capacitance ratio in which the negative supply sensitivity of this configuration offsets the positive supply sensitivity inherent to the VCOs. Li et al. [13] explore an alternative approach using two delay lines characterized by distinct oxide thicknesses and threshold voltages. Consequently, the thermal dependency of the time-delay ratio is predominantly dictated by doping concentration, as opposed to supply voltage variations. Particularly, the susceptibility of all-digital temperature sensors to supply voltage fluctuations has been relatively understudied in the existing literature.

In the meantime, the fundamental physical characteristics related to the thermal coefficient of metal-oxide-semiconductor (MOS) transistors have been investigated as follows. The prior research documented in [14] utilizes the Reverse Short-Channel Effect (RSCE) along with the narrow-channel effect (NCE)-induced threshold voltage ($V_{TH}$) ratios to architect a current reference. The primary objective of this research is to counteract the positive temperature coefficient (TC) of carrier mobility, thereby achieving stabilization of static current amidst temperature fluctuations. In contrast, reference [15] examines the width-dependent threshold voltage ($V_{TH}$), which is influenced by shallow-trench isolation (STI) fringing fields, to mitigate the process-induced $V_{TH}$ variation in a voltage reference. This design principally aims at stabilization of static voltage, with no provisions made for thermal sensing or suppression of supply noise.

In contrast, our design employs a length-dependent threshold voltage ($V_{TH}$) specifically within dynamic delay lines, such as ring oscillators, rather than static current references. This approach serves two primary functions: it facilitates the generation of a digital temperature output by exploiting temperature coefficient (TC) differences between delay lines and Simultaneously mitigates the supply sensitivity issue inherent in oscillator-based temperature sensors. In our research, we emphasize the application of length-dependent $V_{TH}$ as opposed to width-dependent $V_{TH}$, implementing this in dynamic ring oscillators. The TC difference between the two delay lines translates directly into a temperature-sensitive frequency ratio, which is subsequently quantized into a digital output. Furthermore, the intrinsic symmetry of the delay ratio effectively suppresses fluctuations in supply voltage, a critical limitation of single-oscillator sensors that is not addressed in previous works such as those by [14,15].

**Figure 1:**
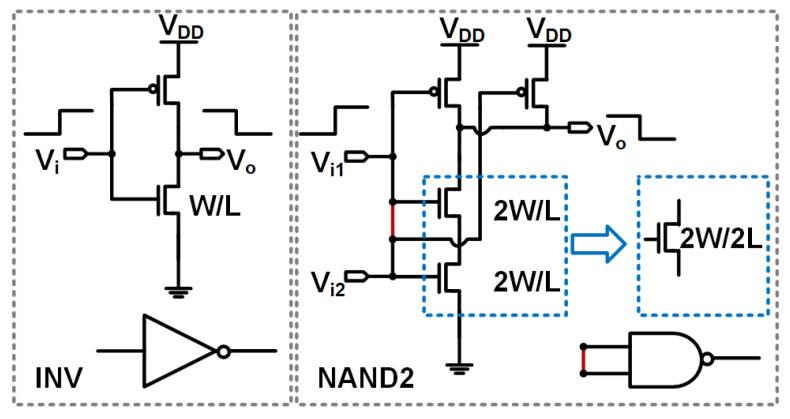
The proposed temperature sensor’s unit cells that form the first (DL1) and second (DL2) delay lines, on the left and right, respectively.

The contributions of this study are outlined as follows: We present an all-digital, synthesizable temperature sensor characterized by a reduction in supply sensitivity. The proposed sensor architecture employs two categories of standard digital logic circuits, namely inverters and NAND gates. The design involves connecting the inputs of the NAND gates together, resulting in different lengths for the equivalent discharging transistors of the NAND and the inverter, thereby inducing differences in their threshold voltages and in the temperature dependence of their propagation delays, as depicted in Figure 1. The NAND delay line is utilized as the timing reference for the inverter delay line. This configuration not only eliminates the requirement for an external clock but also attenuates supply sensitivity by a factor of eight relative to a single delay line. The all-digital temperature sensor operates at a supply voltage of 0.6 V and achieves a conversion energy consumption of 2 nJ with a resolution of 0.2 °C. The sensor was synthesized on a Cyclone IV FPGA, demonstrating similar suppression characteristics of supply sensitivity.

The structure of this manuscript is as follows. Section 2 describes the fundamental operating principle underlying the proposed temperature sensor. The architectural configuration of the circuit is detailed in Section 3. Section 4 provides Monte Carlo simulation results. The measurement results are presented in Section 5. Finally, Section 6 summarizes the conclusions derived from this study.

## 2. Operating Principle

In this section, we conduct an analysis of the thermal and voltage characteristics of threshold voltages in transistors of varying channel lengths.

### 2.1. Physical Principles of Delay Line Thermal Coefficients (TCs)

In the domain of nanoscale CMOS technology, inverter and NAND-based delay lines exhibit distinctive thermal coefficients (TCs) mainly attributed to length-dependent threshold voltage ($V_{TH}$) effects inherent to the 55-nm technology node. This is further exacerbated by the intrinsic physical behavior of transistor stacks during the discharge phase. When the inputs of an NAND gate are shorted, the discharge path is governed by a series stack of two nMOS transistors, as opposed to a single nMOS transistor in the inverter configuration. This structural variation results in two primary physical phenomena that influence $V_{TH}$ and its temperature dependence.

1. Equivalent Channel Length Extension: In an NAND configuration with tied input, the series nMOS stack functions analogously to a single transistor with an effective channel length approximately double that of the inverter nMOS ($L_{\mathrm{NAND}}\approx 2L_{\mathrm{inverter}}$). According to the reverse short-channel effect (RSCE), $V_{TH}$ increases with longer channel length (*L*). Halo implants, which mitigate short-channel effects, decrease $V_{TH}$ in short-channel devices; however, this influence fades as *L* increases, resulting in elevated $V_{TH}$ for extended channels [16,17].

2. Modulation by Body Effect: Within the NAND nMOS series stack, the source terminal of the upper nMOS is connected with the drain of the lower nMOS rather than grounded, as in the inverter’s nMOS. This configuration introduces a nonzero source-body voltage ($V_{SB}$) for the upper transistor, thereby intensifying the body effect—a phenomenon whereby $V_{TH}$ increases with higher $V_{SB}$. This enhanced body effect further increases the effective $V_{TH}$ of the NAND relative to the inverter and alters its thermal response, accentuating the disparity in the thermal dependence of $V_{TH}$ between the two delay lines.

### 2.2. Thermally Dependent Threshold Voltages

A series of simulations were performed on nMOS transistors, specifically those characterized by the model *n*_12_*lprvt*, as depicted in Figure 1. From these simulations, several key observations were derived: (1) the thermal coefficients associated with the threshold voltages under simulation exhibit negative values; (2) an increase in the length of the nMOS transistors from 60 to 120 nm, under constant temperature conditions, results in an elevation of the threshold voltage; (3) a positive correlation exists between the increase in the threshold voltage of the transistor and the reduction of its thermal coefficient as the length increases. The phenomenon outlined in the third observation is explained in [16] and is concisely represented by the relationship $▵V_{TH}\propto -N_{A}/LC_{ox}$, where $V_{TH}$, $N_{A}$, $C_{ox}$, and *L* refer to the threshold voltage, doping concentration, oxide capacitance per unit area, and transistor length, respectively. It is imperative to note that these observations are conditional, dependent on parameters such as the depth of the drain-source junction and the thickness of the channel depletion layer [16]. It is also important to acknowledge that for alternative technologies or device architectures, the threshold voltage may manifest different thermal or voltage coefficients based on variations in the device length [4,13].

### 2.3. Thermally Dependent Propagation Delay Versus Supply Voltage

The propagation delay associated with the discharging of the logic inverter, as illustrated in Figure 1, can be mathematically expressed as

(1) $$t_{dis}=\frac{LC_{L}}{\mu_{n}W_{n}C_{ox}\left(V_{DD}-V_{TH_{n}}\right)}\left(\frac{2V_{TH_{n}}}{V_{DD}-V_{TH_{n}}}+ln\frac{1.5V_{DD}-2V_{TH_{n}}}{0.5V_{DD}}\right)$$

Similarly, the charging propagation delay can be deduced as:

(2) $$t_{charge}=\frac{LC_{L}}{\mu_{p}W_{p}C_{ox}\left(V_{DD}-V_{TH_{p}}\right)}\left(\frac{2V_{TH_{p}}}{V_{DD}-V_{TH_{p}}}+ln\frac{1.5V_{DD}-2V_{TH_{p}}}{0.5V_{DD}}\right)$$

Equations (1) and (2) correspond to the study by [18], where the symbols $V_{DD}$, $C_{L}$, μ, and *W* denote the supply voltage, load capacitance, carrier mobility, and transistor width, respectively. These parameters constitute the propagation delay of a single inverter. The initial term $2LC_{L}V_{TH}/\left[\mu_{n}W_{n}C_{ox}{\left(V_{DD}-V_{TH}\right)}^{2}\right]$ in Equation (1) arises from the discharging process when the nMOS transistor operates in the saturation region. The subsequent term corresponds to the linear region operation, applicable when $V_{o}<V_{i}-V_{TH}$. The temperature dependence of the threshold voltage, $V_{\mathrm{TH}}$, is represented by the relation $V_{\mathrm{TH}(T)}=V_{TH0}-\alpha T$, where α is a positive coefficient and $V_{TH0}$ represents the threshold voltage at absolute zero temperature. The carrier mobility temperature coefficient (μ) is negative, expressible as $\mu \propto T^{-\gamma }$, with γ approximately equal to 1.5.

### 2.4. Power Supply Sensitivity Reduction

Initially, as indicated in reference to [4], we assume that

(3) $$t_{dis}=\frac{2LC_{L}V_{TH}}{\mu WC_{ox}{\left(V_{DD}-V_{TH}\right)}^{\beta }}$$

According to the findings presented by [4], the parameter β is approximately equal to 1, with the subscript *n* being excluded. Upon the introduction of a secondary delay line characterized by a distinct length, the ratio of their propagation delays can be mathematically represented as follows:

(4) $$\frac{t_{dis2}}{t_{dis1}}=\frac{V_{DD}-V_{TH1}}{V_{DD}-V_{TH2}}$$

Assuming that $C_{L},W,L$ are equivalent for both oscillators and that β equals 1, an increase in the supply voltage by $\Delta V_{DD}$ results in a modification of Equation (3) as follows:

(5) $$\Delta t_{dis}=\frac{2LC_{L}V_{TH}\Delta V_{DD}}{\mu WC_{ox}\left(V_{DD}-V_{TH}\right)\left(V_{DD}+\Delta V_{DD}-V_{TH}\right)}$$

To solve the supply sensitivity, we have to divide Equation (5) by Equation (3) and obtain:

(6) $$\frac{\Delta t_{dis}}{t_{dis}}=\frac{\Delta V_{DD}}{V_{DD}+\Delta V_{DD}-V_{TH}}$$

In a similar manner, the sensitivity of supply for the ratio of two delays with varying thresholds, as described in Equation (4), is expressed as follows:

(7) $$\frac{\Delta (t_{dis1}/t_{dis2})}{t_{dis1}/t_{dis2}}=\frac{\Delta V_{DD}\left(V_{TH1}-V_{TH2}\right)}{\left(V_{DD}+\Delta V_{DD}-V_{TH2}\right)\left(V_{DD}-V_{TH2}\right)}$$

Upon examination of Equations (6) and (7), it can be deduced that Equation (7) exhibits a reduced sensitivity to variations in supply, quantifiably by a factor denoted as *F*.

(8) $$F=(V_{DD}-V_{TH2})/(V_{TH1}-V_{TH2})$$

**Figure 2:**
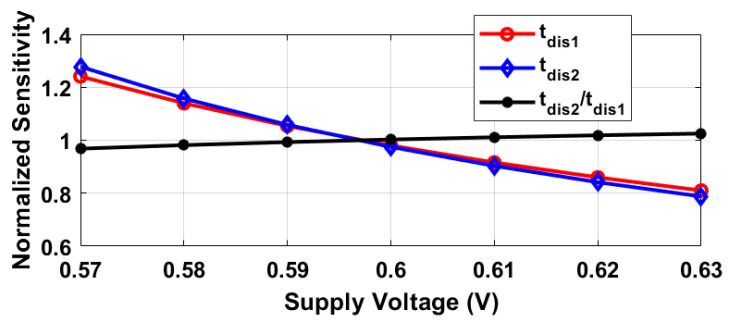
Simulated supply sensitivity for 60 nm and 120 nm device lengths, normalizing delay and ratio sensitivities to their averages. Using $V_{TH}$ from Figure 3 and Equations (3) and (4), an 8-fold reduction in supply sensitivity is noted for $t_{dis2}/t_{dis1}$ compared to $t_{dis1}$ or $t_{dis2}$ alone.

**Figure 3:**
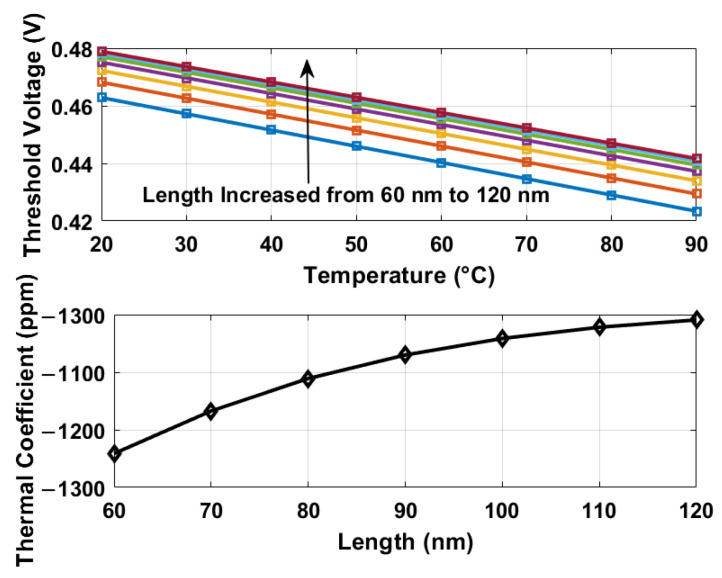
Simulated threshold voltages for nMOS lengths (60–120 nm) and thermal coefficients. Thermal Coefficient $\left(\mathrm{ppm}{/}^{{}^{\circ}}C\right)=\frac{\left(V_{max}-V_{min}\right)}{V_{nom}\cdot \Delta T}\times 10^{6}$, where ΔT is the temperature range; $V_{max},V_{nom},V_{min}$ are the maximum, nominal, and minimum voltages.

Given the parameters $V_{DD}\approx 0.6$, $V_{TH}\approx 0.4$, and $V_{TH1}-V_{TH2}\approx 0.025$, the calculated suppression factor is approximately 8. To validate these assumptions, Figure 2 illustrates the relationship between supply voltage variations and the discharging times for two different delay configurations, alongside their ratio as described by Equation (4). This analysis utilizes simulated threshold voltages presented in Figure 3, as well as Equations (3) and (4), applied to two devices with channel lengths of 60 nm and 120 nm, respectively. When comparing $t_{dis1}$ and $t_{dis2}$, both demonstrate a supply sensitivity of roughly 27%. However, the ratio $t_{dis2}/t_{dis1}$ (as per Equation (4)) exhibits a significant reduction in supply sensitivity, measured to be 3.2%, indicating an eightfold decrease.

The thermal coefficient of $V_{TH}$ (α), defined as the rate of variation of $V_{TH}$ with temperature, exhibits a less negative trend as the channel length increases. Short-channel transistors possess elevated dopant concentrations, which increase the temperature sensitivity of $V_{TH}$, resulting in more negative α values. In contrast, longer channels exhibit diminished dopant concentration-related thermal sensitivity, yielding α values with less negativity [16]. These variations in $V_{TH}$ and α directly influence the propagation delay’s TCs: as described in Equation (1), the discharge delay ($t_{dis}$) is inversely related to ($V_{DD}-V_{TH}$) ($t_{dis}\propto 1/\left(V_{DD}-V_{TH}\right)$). Consequently, the disparate values α of the two delay lines establish a predictable TC ratio with respect to their propagation delays, thereby facilitating the isolation of temperature information while mitigating supply voltage perturbations.

In the context of propagation delay without simplification, as expressed in Equation (1), the sensitivity to supply voltage is influenced by the relative magnitudes of $V_{DD}$ and $V_{TH}$, which are subject to variation as the technology node advances. To effectively mitigate this supply sensitivity, it is essential to ensure that the difference $V_{DD}-V_{TH2}$ substantially exceeds that of $V_{TH1}-V_{TH2}$, irrespective of the variations in β as defined in Equation (3). While the sensitivity of the pMOS charging delay may not precisely mirror that of the nMOS, ensuring that the ratio, as depicted in Equation (8), exceeds unity is sufficient to suppress its supply sensitivity.

## 3. Circuit Architecture

### 3.1. Thermal Dependent Digital Outputs

**Figure 4:**
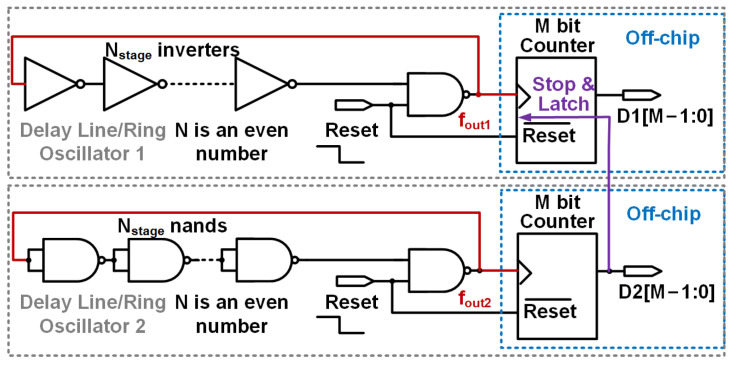
The proposed dual-oscillator temperature sensor features two ring oscillators: Oscillator 1 (DL1) with N-stage inverters and Oscillator 2 (DL2) with N-stage NAND gates, both with even N, plus two off-chip M-bit counters. Oscillator 1 and 2 produce $f_{\mathrm{out}1}$ and $f_{\mathrm{out}2}$, respectively, synchronized by a reset signal, and counted to outputs D1[M−1:0] and D2[M−1:0].

Figure 4’s logic-cell-based ring oscillators convert the thermal-dependent delay from Equations (3) and (4) into digital outputs.

At the moment the second line (DL2)’s output counts the designated value D0, which is kept for the entire temperature range, the digital outputs of the first line, represented as DTS(T), are as follows:

(9) $$DTS\left(T\right)=\frac{f_{out1}}{f_{out2}}\cdot D0$$

Combining Equations (4) and (9), one obtains

(10) $$DTS\left(T\right)=\frac{V_{DD}-V_{TH_{0,1}}+\alpha 1\cdot T}{V_{DD}-V_{TH_{0,2}}+\alpha 2\cdot T}\cdot D0$$

The relationship among Equations (4), (9) and (10) can be further explained as follows. The two delay lines (DL1 and DL2) utilize transistors of varying lengths, resulting in distinct thermal coefficients ($\alpha_{1}/\alpha_{2}$) for their threshold voltages ($V_{TH}$) as shown in Figure 3 and detailed in Section 2.1. As derived in Equation (4), the ratio of their propagation delays ($t_{dis2}/t_{dis1}$) is given by ($V_{DD}-V_{TH1}$)/($V_{DD}-V_{TH2}$); this ratio serves the dual purpose of retaining thermal information and mitigating supply noise, which is crucial for stable temperature sensing. Given that the oscillation frequency ($f_{out}$) is inversely proportional to the propagation delay ($t_{dis}$), that is, $f_{out}\propto 1/t_{dis}$, the frequency ratio ($f_{out1}/f_{out2}$) corresponds to the propagation delay ratio ($t_{dis2}/t_{dis1}$). During sensor operation, the counter latches the fixed output ($D_{0}$) of DL2. Subsequently, the digital output $D_{TS}\left(T\right)$ of DL1 is calculated as the product of the frequency ratio ($f_{out1}/f_{out2}$) and $D_{0}$, as formally expressed in Equation (9). By substituting the propagation delay ratio relationship from Equation (4) into Equation (9), we derive Equation (10), which explicitly relates the thermal coefficients of the threshold voltage ($\alpha_{1}/\alpha_{2}$) to the digital output $D_{TS}\left(T\right)$, thus establishing a direct connection between these two principal equations.

In summary, the substitution of $V_{TH}=V_{TH0}-\alpha T$ into the frequency ratio results in Equation (10), which directly quantifies the ratiometric thermal signal into a digital output. By expressing the threshold voltage and its associated thermal coefficient as $V_{TH}=V_{TH_{0}}-\alpha T$, the integer value of DTS(T) becomes proportional to the thermal coefficients of both transistors. For Equation (10) to manifest thermal dependency, it is imperative that the two ring oscillators incorporate transistors with varied lengths, each exhibiting distinct thermal dependencies in their threshold voltages. To achieve this configuration, the nand standard cell is selected as illustrated on the right side of Figure 1, where both inputs of the nand gates are interconnected. This configuration effectively combines the two transistors encompassed within the blue dotted box depicted in Figure 1 into a single transistor approximately twice the length. The design objectives are to construct temperature sensors that utilize standard cells and to ensure synthesizability of the design. Alternatively, employing another longer length inverter line is feasible.

### 3.2. Resolution of the Temperature Sensor

The resolution of the proposed temperature sensor can be established in the following manner. By calculating the variation in DTS(T) as described by Equation (10) when the temperature increases from *T* to T+▵T, and specifically when ▵T=1 °C, the resultant value is determined.

(11) $$DTS\left(T+▵T\right)-DTS\left(T\right)=\frac{V_{eff1}}{V_{eff2}}\cdot \frac{\alpha 1-\alpha 2}{{\left(1+\alpha 2\cdot T\right)}^{2}}\cdot D0$$

which is the change in Digital Numbers (DN) for every 1 °C temperature rise. $V_{eff}=V_{DD}-V_{TH}$. The inverse of Equation (11) represents the resolution in °C/DN.

### 3.3. Limitations of Supply Voltage

**Figure 5:**
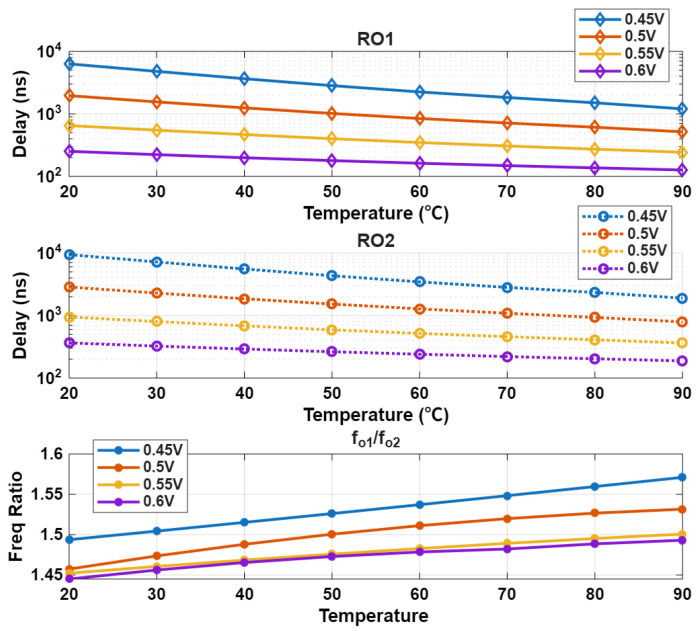
Normalized oscillation frequency of RO1/RO2 vs. Temperature (**Top**, **Middle**) and frequency ratio RO1:RO2 vs. Temperature (**Bottom**).

As depicted in Figure 3, the threshold voltage ($V_{\mathrm{TH}}$) of the nMOS transistor is approximately 480 mV. For MOSFETs, $V_{\mathrm{TH}}$ denotes the minimum gate-source voltage ($V_{\mathrm{GS}}$) required to trigger conduction in the linear region; thus, when $V_{\mathrm{GS}}$ is below or only slightly above this 480 mV threshold, the nMOS transistor operates in the subthreshold (or weak inversion) region. Notably, the ring oscillators (ROs) of the proposed design may still maintain functionality even in this subthreshold regime. To identify the minimum usable supply voltage ($V_{\mathrm{DD}}$)—a critical parameter for low-power applications—we performed simulations using Cadence Virtuoso, and the corresponding results are presented in Figure 5. The top subplot in Figure 5 presents the normalized oscillation frequency response of ring oscillator 1 (RO1, red diamond) and ring oscillator 2 (RO2, black circle) as $V_{\mathrm{DD}}$ fluctuates from 0.45 V to 0.6 V, versus the temperature range between 20 and 90 °C. The bottom subplot displays the frequency ratio (RO1/RO2), when $V_{\mathrm{DD}}$ decreases from 0.6 V to 0.45 V. Below 0.4 V, RO1 and RO2 struggle to sustain oscillation, and below 0.35 V, approximately 130 mV below the nMOS $V_{\mathrm{TH}}$ of 480 mV, both oscillators fail to function normally as the transistors turn off. This transition defines the minimum functional $V_{\mathrm{DD}}$ boundary for our design, likely at 0.45 V. However, on the chip, the oscillation frequency signal—originally associated with the aforementioned $V_{\mathrm{DD}}$ is converted to the chip’s 1.2 V DVDD using a standard low-to-high level shifter [19]. When the input voltage falls below 0.6 V, this low-to-high conversion fails to operate normally. Thus, while our design specifies a minimum supply voltage of 0.6 V for external operation, the internal working voltage of the chip can actually be reduced to 0.4 V.

### 3.4. Phase Noise Analysis

The spectral density of phase noise associated with the ring oscillator is expressed into its constituent white noise and flicker noise components, denoted as $S_{\Phi ,\mathrm{white},\mathrm{ring}}\left(\Delta f\right)$ and $S_{\Phi ,1/f,\mathrm{ring}}\left(\Delta f\right)$, respectively. These components are illustrated in Figure 1 and can be mathematically represented as outlined in [20].

(12) $$S_{\Phi ,\mathrm{white},\mathrm{ring}}\left(\Delta f\right)=\frac{f_{\mathrm{osc}}^{2}}{\Delta f^{2}}\left\{\frac{1}{2I_{D}^{2}}[S_{I}\left(\Delta f\right){|}_{\mathrm{NMOS}}+S_{I}\left(\Delta f\right){|}_{\mathrm{PMOS}}]+\frac{2kT}{I_{D}V_{\mathrm{DD}}}\right\}$$

(13) $$S_{\Phi ,1/f,\mathrm{ring}}\left(\Delta f\right)=\frac{f_{\mathrm{osc}}^{2}}{4MI_{D}^{2}\Delta f^{2}}[S_{1/f}\left(\Delta f\right){|}_{\mathrm{NMOS}}+S_{1/f}\left(\Delta f\right){|}_{\mathrm{PMOS}}]$$

In the context of the ring oscillator, $f_{\mathrm{osc}}$ represents the primary oscillation frequency. A decrease in $f_{\mathrm{osc}}$ is correlated with a reduction in phase noise. The parameter *M* denotes the count of inverter stages within the ring oscillator, where a larger *M* is associated with a decrease in flicker noise. The drain current, denoted as $I_{D}$, corresponds to the MOS transistors (both NMOS and PMOS) present in the inverter stages of the ring oscillator. An increase in $I_{D}$ leads to a reduction in noise, given that noise inversely scales with $1/I_{D}^{2}$. The variable Δf signifies the offset frequency from $f_{\mathrm{osc}}$, and flicker noise becomes predominant at lower values of Δf. As flicker noise is proportional to $S_{1/f}\propto \frac{1}{W\cdot L}$, a reduction in the dimensions of nMOS and pMOS transistors results in increased flicker noise.

## 4. Monte Carlo Simulation Results

**Figure 6:**
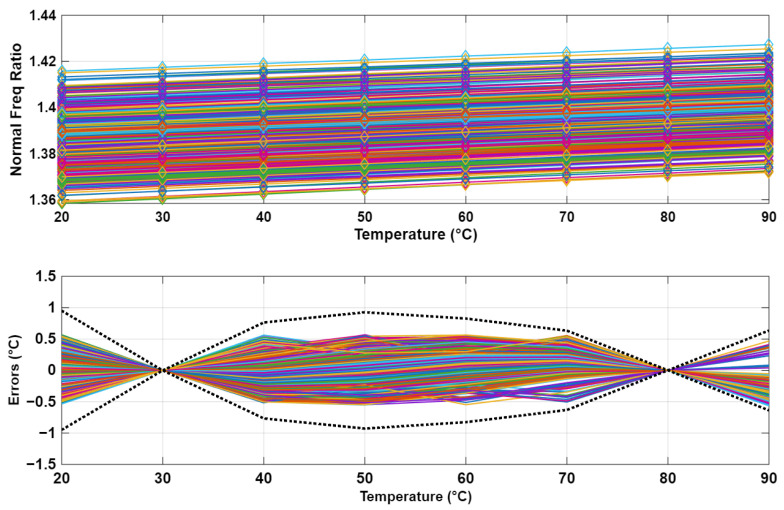
Raw digital outputs (top) and errors from 500 Monte Carlo simulations after a global linear fit and calibration at 30 and 80 °C per sensor (bottom). The black dotted lines in the bottom figure represent the 3 σ errors.

To evaluate the sensitivity of the proposed temperature sensors to mismatches, a set of 500 Monte Carlo simulations was executed that incorporated device-to-device variability. These simulations were based on the circuit schematic expressed in Figure 1 and Figure 4, and the Equation (10). The resultant data from these simulations are presented in Figure 6, following a two-point calibration procedure for each sensor, coupled with a global linear fit methodology. The 3 σ errors derived from these 500 Monte Carlo simulations are effectively restricted within the ±1.2 °C range.

In the absence of either two-point or one-point calibration, the uncalibrated digital outputs presented in Figure 6 demonstrate significant errors that exceed the specified temperature range of 70 °C. This discrepancy arises because, among the various uncalibrated curves, those with comparatively lower values yield simulated digital outputs at 90 °C that are lower than the measured values at 20 °C for the curves with relatively higher values.

This study employs a two-point calibration methodology to enhance precision to ±1.2 °C. Within the scope of thermal management of VLSI, a deviation of the relative temperature measurement of 3–5 °C is generally considered adequate. An industry standard approach comprises two complementary elements: (1) a single, highly precise temperature sensor, typically of the BJT or resistor variety, subjected to precise trimming calibration during manufacturing; and (2) multiple compact sensors with lower accuracy (3 to 5 °C deviation) strategically placed near thermal hotspots. This inaccuracy of 3 to 5 °C can be obtained by one-point calibration (self-calibration at room temperature, for example). These compact sensors are capable of self-calibration by utilizing the highly precise sensor as a reference during system initialization, thus refining their output based on its reliable temperature data [21]. The design in work would serve as the compact sensors with 3–5 °C accuracy between 20–90 °C upon on-point calibration at room temperature.

## 5. Measurement Results and Discussion

### 5.1. 55 nm CMOS Prototype

**Figure 7:**
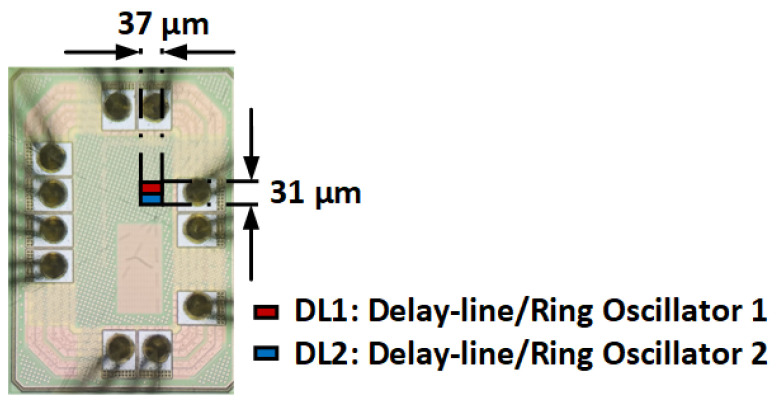
Micrograph of the proposed temperature sensor.

The prototype of the proposed temperature sensor, fabricated using 55 nm CMOS technology, occupies an area of 37 × 31 $\mu m^{2}$ as depicted in Figure 7.

**Figure 8:**
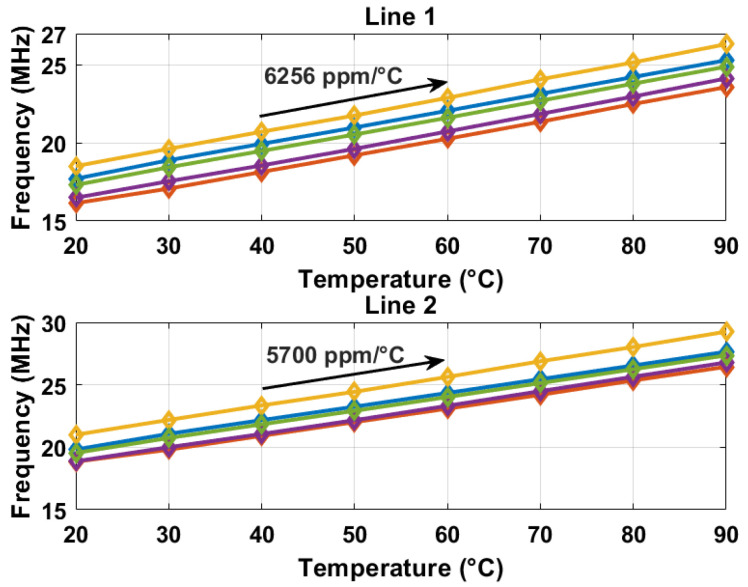
Oscillation frequencies measured for two types of delay lines on five chips.

**Figure 9:**
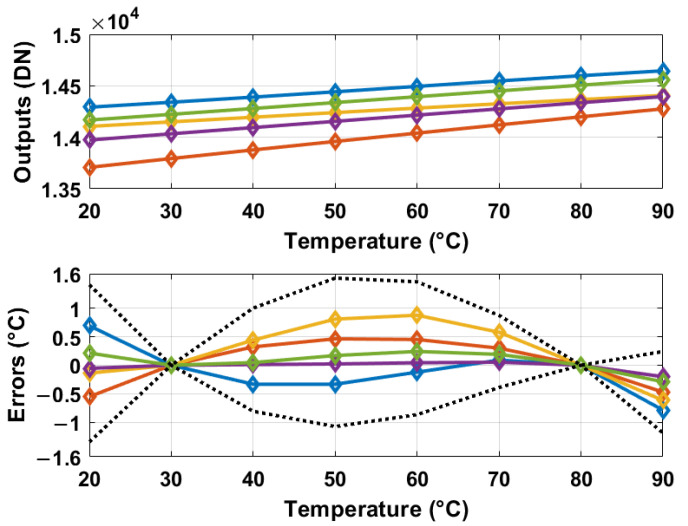
Measured digital outputs and errors for five temperature sensors. Errors are based on a two-point calibration at 30 and 80 °C for each sensor and a global linear fit. Dotted lines indicate 3 σ deviations.

**Figure 10:**
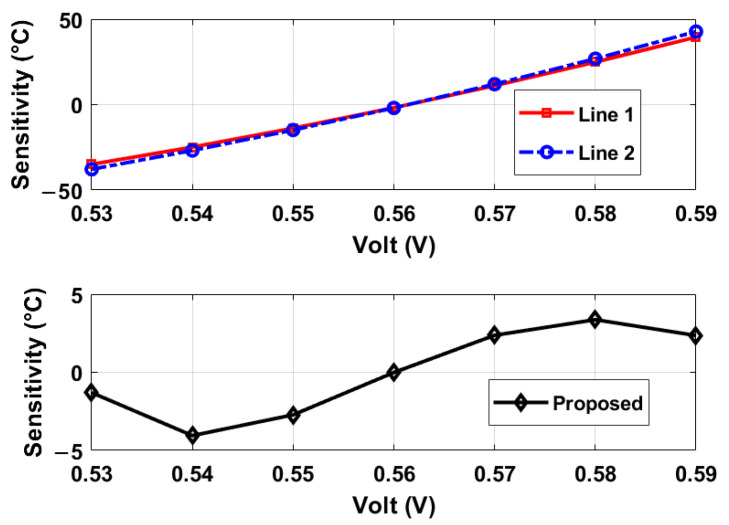
Supply sensitivity-induced errors were measured for delay lines 1, 2, and the proposed temperature sensor at 20 °C. Delay lines 1 and 2 show a supply sensitivity of about 0.8 °C/mV, while the proposed method reduces this by approximately 8 times to 0.08 °C/mV.

**Figure 11:**
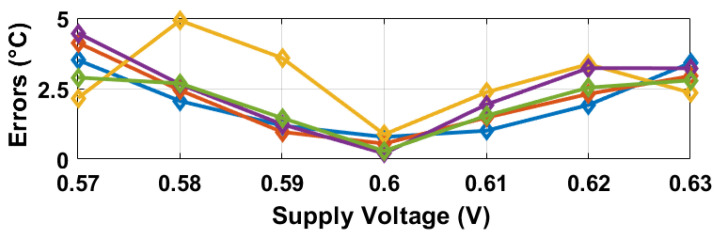
Measured supply sensitivity errors for five chips at 20 °C.

The oscillation frequencies of both delay lines were measured as a function of temperature across five different chips, as illustrated in Figure 8. The temperature reference employed was a commercially available temperature sensor [22]. The sensor achieves a resolution of 0.2 °C by adjusting the stop and latch counting number D0 in Equation (11) to 14,660 (DN), in conjunction with the α1 and α2 values of $6.256\times 10^{-3}$ and $5.7\times 10^{-3}$ respectively, derived from Figure 8. For delay line 2 (DL2), this setting results in a minimum conversion time of approximately 14,660/19.6 MHz ≈ 750 µs. To maintain a margin, a D0 value of 16,000 (DN) was utilized, and the digital outputs from delay line 1 (DL1) were recorded as demonstrated in Figure 9. Post two-point calibration at 30 and 80 °C for each chip, alongside a global first-order fitting, the inaccuracy of the five measured sensors was confined within ±1 °C over the temperature range of 20 °C to 90 °C. This range is suitable for VLSI thermal management applications. The calibration approach is analogous to that described in [23]. Figure 10 illustrates the measured supply sensitivity for delay lines 1, 2, and the proposed temperature sensor, indicating a tenfold reduction in supply sensitivity, corresponding to the simulation findings in Figure 2, which indicate an approximately eightfold reduction in supply sensitivity through the proposed methodology. The supply sensitivity-induced errors across five test chips, when the supply voltages ranged from 0.57 V to 0.63 V, are presented in Figure 11, revealing a measured supply sensitivity of approximately 0.08 °C/mV. The sensor’s 1 σ root mean square (rms) noise, computed from the rms of multiple digital outputs, is approximately 0.8 DN, translating to 0.16 °C. As with all oscillator-based temperature sensors, enhanced resolution can be achieved by extending the conversion time, specifically in this study by increasing D0. However, this will result in increased energy consumption per conversion as the conversion time extends. The resolution’s lower limit is constrained by the noise, quantified at 0.16 °C in this study.

### 5.2. FPGA-Synthesized Temperature Sensor

**Figure 12:**
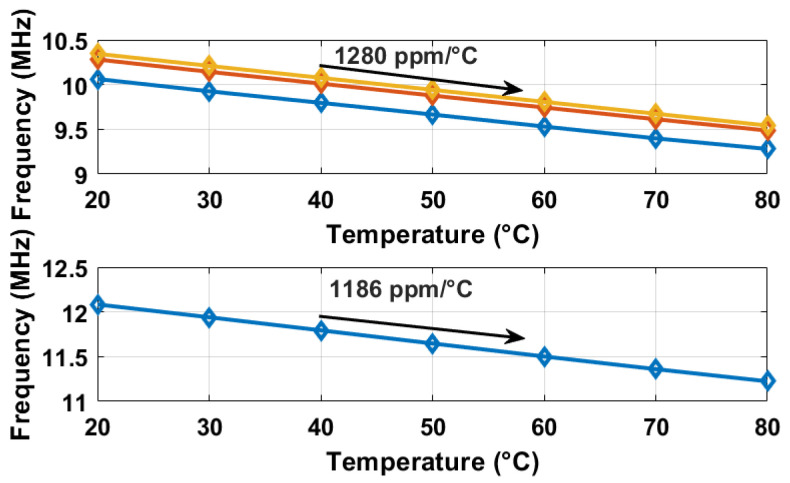
Measured oscillation frequencies for the two types of oscillators, and the first type was implemented three times, and the second type once, as the timing reference, on the same FPGA.

**Figure 13:**
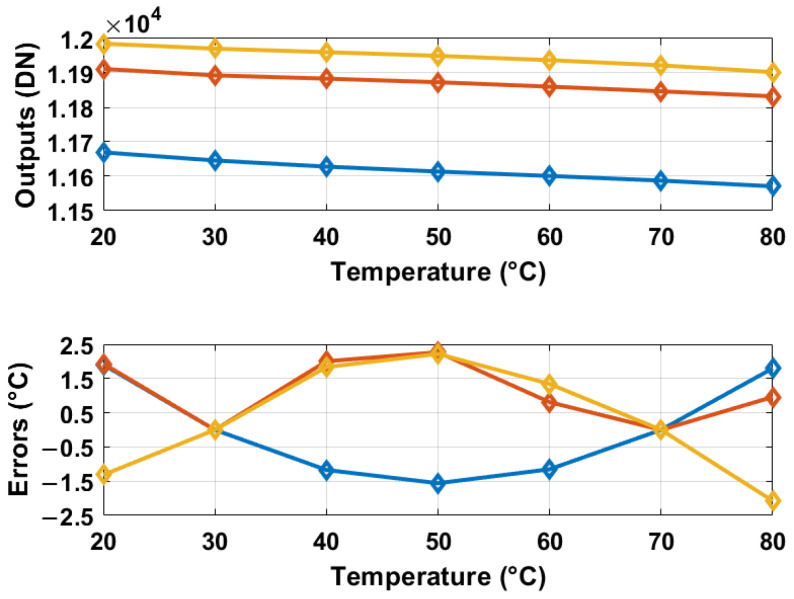
Measured digital outputs and errors of three FPGA-based temperature sensors after two-point calibration at 30 and 70 °C and 3rd order global curve fit.

**Figure 14:**
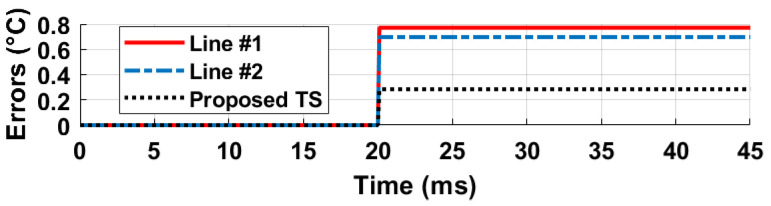
Measured dynamic supply sensitivity-induced errors, for delay line 1, 2 and the proposed temperature sensor, for an approximately 10 mV voltage rise at 20 ms on the cyclone IV FPGA. An approximately 3 times reduction in supply sensitivity is observed in the proposed temperature sensor (TS).

The temperature sensor under consideration, depicted in Figure 4, is implemented on a 60 nm Cyclone IV FPGA. To prevent the removal of inverter/NAND gates during optimization processes, the instruction (*keep = 1*) is incorporated within the Verilog code. The configuration closely resembles that of the previously discussed IC prototype. The experimentally derived oscillation frequencies, digital outputs, and associated errors as a function of temperature are presented in Figure 12 and Figure 13, respectively. Additionally, the dynamic supply sensitivity-induced errors are displayed in Figure 14. Figure 13 reveals that the uncalibrated outputs, from three different placed and route on the same FPGA, demonstrate consistent thermal coefficients; however, they vary in digital values at identical temperatures, likely due to process variations. In particular, after two-point calibration, the error is measured ±2.5 °C within the temperature range of 20 °C to 80 °C. Differences between the prototypes based on the IC and the FPGA can be discerned from the measurement results, as follows. (i) The thermal coefficient of the measured oscillation frequency is negative in Figure 12, in contrast to the positive coefficients observed in Figure 8. This discrepancy is attributed to the reduced supply voltage $V_{DD}$ in Figure 8 relative to Figure 12, when considered against $V_{TH}$. (ii) The FPGA-based temperature sensor exhibits greater curvature in its outputs as shown in Figure 12, similar to the behavior reported in [12]. (iii) Both implementations demonstrate the capability to mitigate supply sensitivity for the proposed temperature sensor, in comparison to a standalone delay line. Detailed explanations of observation (i) are as follows: (1) The oscillation frequency ($f_{\mathrm{out}}$) of a ring oscillator is inversely related to the propagation delay ($t_{dis}$) of its unit logic cells, according to the relationship $f_{\mathrm{out}}\propto 1/t_{dis}$. Consequently, the thermal coefficient of $f_{\mathrm{out}}$ is opposite in sign to that of $t_{dis}$, necessitating an analysis of the temperature dependence of $t_{dis}$. (2) The threshold voltage ($V_{\mathrm{TH}}$) possesses a negative thermal coefficient, mathematically described by $V_{\mathrm{TH}}=V_{\mathrm{TH}0}-\alpha T$ where α>0. As temperature rises, $V_{\mathrm{TH}}$ decreases, potentially reducing $t_{dis}$, as per Equation (3). Moreover, the carrier mobility (μ) exhibits a negative thermal coefficient, denoted as $\mu \propto T^{-\gamma }$ with γ≈1.5. An increase in temperature results in a decline in μ, which tends to elevate $t_{dis}$, as indicated by Equation (3). (3) In Figure 8’s IC implementations, $V_{\mathrm{DD}}$ is marginally greater than $V_{\mathrm{TH}}$. Here, the reduction of $V_{\mathrm{TH}}$ with increasing temperature predominates, leading to a decrease $t_{dis}$ and a increase in $f_{\mathrm{out}}$; therefore, the thermal coefficient of $f_{\mathrm{out}}$ is positive. In Figure 11’s FPGA, $V_{\mathrm{DD}}$ significantly exceeds $V_{\mathrm{TH}}$. In this scenario, the reduction in μ with temperature is predominant, resulting in increased $t_{dis}$ and decreased $f_{\mathrm{out}}$; therefore, the thermal coefficient of $f_{\mathrm{out}}$ is negative.

The proposed temperature sensor is evaluated against state-of-the-art works, as demonstrated in Table 1. This study introduces an entirely digital, synthesizable temperature sensor exhibiting an eightfold reduction in supply sensitivity. Furthermore, it operates at a supply voltage as low as 0.6 V, adjustable within the range of 0.6 V to 1.2 V. Additionally, it features relatively low RFOM and area metrics. While [13] incorporates specialized analog circuitry and custom multi-threshold MOSFETs to achieve superior supply sensitivity, our all-digital design is completely synthesizable using standard logic cells, eliminating the need for specialized devices or process customization, thereby facilitating straightforward integration into System-on-Chip (SoC) platforms. Its operational range of 0.6 V to 1.2 V addresses ultra-low-power requirements for IoT and edge computing applications, contrasting with [13]’s 0.95 V supply voltage, which results in significant power overhead, thus enhancing its applicability for mass-produced digital systems.

## 6. Conclusions

We present a 0.6-V, fully digital, synthesizable temperature sensor designed to mitigate supply sensitivity. The sensor achieves an eightfold reduction in supply sensitivity by employing two distinct types of standard logic delay lines that possess different thermal coefficients for their threshold voltages as a result of variations in transistor lengths. This device is fabricated using the 55 nm CMOS technology and occupies an area of 0.0011 ${\mathrm{mm}}^{2}$. It demonstrates an inaccuracy of within ±1 °C across the temperature range of 20–90 °C. At a conversion time of 0.8 ms and a resolution of 0.2 °C, the sensor’s energy consumption is quantified at 2 nJ per conversion. If it is integrated onto a chip, the 14-bit counter is projected to occupy an area of 20 × 150 $\mu m^{2}$, with its power consumption dependent upon the frequency of the input signal and the design architecture.

## Figures and Tables

**Table 1:** Performances Compared with the State-of-the-art Works.

Parameters	This Work	This Work	[13]	[24]	[12]
Year	2023	2023	2021	2020	2019
Technology	55	FPGA	130	350	FPGA
(nm)					
**All-digital?**	**Yes**	**Yes**	No	No	Yes
**Synthesized?**	No	**Yes**	No	No	Yes
**Supply**					
**Sensitivity**	0.08	0.08	0.014	N/A	N/A
(°C/mV)					
Calibration	2-point	2-point	2-point	2-point	2-point
Method	+linear	+linear	+linear	+linear	+poly
**Supply (V)**	**0.6**	1.2	3.3	0.95	1.2
**Area (${\mathrm{mm}}^{2}$)**	**0.0011**	80 inv	0.019	0.004	74 inv
PP IA ${\left({}^{\circ}C\right)}^{\;1}$	1	2.5	0.84	1.35	0.9
Min (°C)	20	20	0	0	−20
Max (°C)	90	80	80	90	100
Rel. IA ^1^	1.42	4.17	1.05	1.5	1.1
Conversion	0.8	0.24	59	0.1	1
Time (ms)					
Power (µW)	2.5	12	0.196	5	90
Energy (nJ) per conversion	2	2.88	11.56	0.5	90
Resolution (°C)	0.2	0.5	0.1	0.1	0.05
${\mathrm{RFOM}}^{2}$ $\left(\mathrm{nJ}\times K^{2}\right)$	0.08	0.72	0.12	0.01	0.225

^1^ The relative inaccuracy (Rel. IA) is defined as 100 · PP IA/Specified temperature range where PP IA is the worst-case inaccuracy (IA) over a specified temperature range. The RFOM^2^ is calculated as the Energy/Conversion·Resolution^2^ and is a measure of energy efficiency in reference to its resolution [25].

## Data Availability

The original contributions presented in this study are included in the article. Further inquiries can be directed to the corresponding author.
